# A virtual program to teach pain coping skills to dyads of caregivers and Veterans with dementia or mild cognitive impairment: Preliminary quantitative and qualitative findings

**DOI:** 10.1017/S1478951526102144

**Published:** 2026-04-08

**Authors:** Debra K. Weiner, Ina Engel, Megan Hamm, Coleen Cardamone, Michelle I. Rossi, Subashan Perera, Katherine Ramos, Laura S. Porter

**Affiliations:** 1 University of Pittsburgh School of Medicine, Pittsburgh, PA; 2 Geriatric Research, Education and Clinic Center, Veterans Affairs (VA) Pittsburgh Healthcare System, Pittsburgh, PA; 3 Geriatrics and Extended Care, VA Pittsburgh Healthcare System, Pittsburgh, PA; 4 University of Pittsburgh Graduate School of Public Health, Pittsburgh, PA; 5 Department of Psychiatry & Behavioral Sciences, Duke University Medical Center, Durham, NC; 6 Veterans Rural Health Resource Center-Salt Lake City, Salt Lake City, UT

**Keywords:** Caregiver-patient dyads, veterans, pain, dementia, pain coping

## Abstract

**Objectives.:**

To evaluate the feasibility and preliminary efficacy of a clinical program designed to teach informal caregivers of older Veterans with pain and mild-to-moderate dementia or mild cognitive impairment (MCI), pain management, pain coping and pain communication skills.

**Methods.:**

Twenty caregivers of older Veterans with pain and dementia or MCI and the Veterans themselves participated in a 5-session program taught by trained Veterans Affairs (VA) clinicians. All sessions were conducted remotely using video-technology, with caregivers and Veterans. Two sessions were conducted with individual Veteran-caregiver dyads, and three sessions were conducted with caregiver groups. Caregivers and Veterans completed baseline and post-intervention measures. Qualitative interviews of 10 caregivers who completed the program were also conducted and focused on identifying themes related to caregiving for their loved ones with pain and dementia and related to participating in the program.

**Results.:**

The program was well received and almost all caregivers identified videoconferencing as the preferred venue for participating in such a program. They most valued learning about dementia and participating with other caregivers. Pre-post analyses revealed significant improvements in perceived caregiving competence and self-efficacy for managing pain. Challenges encountered included scheduling related to caregivers’ multiple competing responsibilities and lack of familiarity with tele-conferencing technology.

**Significance of results.:**

Patients with pain and mild to moderate dementia or MCI have been relatively ignored in current literature. Our preliminary findings suggest that a program delivered by trained healthcare professionals to caregivers and Veterans using tele-conferencing could benefit caregivers.

## Introduction

Chronic pain and dementia are sources of immeasurable suffering and healthcare resource utilization, and the projected demographic shift of our population predicts a steady increase in both over the foreseeable future (Hunt et al. 2015; Fang et al. 2025). Older adults with dementia pose unique pain management challenges, including difficulty in assessing pain and in applying safe and effective pain management strategies that require intact cognitive skills such as cognitive behavioral therapy and mindfulness meditation (Broderick et al. 2016; Morone et al. 2016). As a result, their caregivers may experience heightened stress and its associated morbidities (Chiao et al. 2015).

Most of the literature related to people with pain and dementia has focused on the development of behavioral pain assessment tools for those with advanced dementia who are nonverbal (Warden et al. 2003; Zwakhalen et al. 2006; Malara et al. 2016). Those with mild to moderate dementia have been relatively ignored (Wright et al. 2016). Dyadic interventions (i.e., caregiver-patient) have been developed for cognitively intact patients with pain (Smith et al. 2019), and we have published the results of a single-arm pilot study in dyads of caregivers and verbal patients with dementia who have non-cancer pain (Porter et al. 2022). The intervention focused on teaching caregivers pain coping skills, pain distraction techniques, and other non-pharmacological pain management strategies. The 5-session hybrid intervention (i.e., sessions offered in-person or by phone) was tested in 11 caregiver-patient dyads, and found to be feasible and associated with a high level of satisfaction (Porter et al. 2022).

The purpose of the feasibility study described here was to corroborate, extend, and adapt these findings to Veterans, in whom the prevalence of dementia is greater than in the general community because of risk factors such as traumatic brain injury and post-traumatic stress disorder (Raza et al. 2021). As with the prior study, we included participants with either dementia or mild cognitive impairment (MCI). We modified the prior study’s design in several ways. First, we targeted Veteran-caregiver dyads as compared with a general community sample in the prior study. Second, we delivered the intervention entirely using a virtual format to accommodate many families’ rural location and consequent challenges with attending in person appointments, whereas in the prior study only some of the intervention was delivered virtually. Third, the intervention sessions included a mixture of caregiver-only groups and individual caregiver-patient dyads, versus individual caregiver-patient dyads alone in the prior study because of the value of informal dementia caregiver groups that has been demonstrated by others (Lauritzen et al. 2012). Finally, the intervention sessions were delivered by clinical staff experienced in working with older adults, but who had no background or prior training in delivering pain coping skills interventions.

## Methods

All participating Veterans were established patients in the Veterans Affairs (VA) Pittsburgh Healthcare System’s (VAPHS) Geriatric Evaluation and Management (GEM) Clinic, the VAPHS Teledementia Clinic, or the VAPHS Pain Clinic. No randomization took place. Caregivers were approached by their Veteran’s clinic staff about the possibility of participating in the project. Institutional Review Board approval was not required.

Caregiver-Veteran dyads were selected to participate if the Veteran: 1) had an electronic medical record (EMR)-documented diagnosis of dementia or mild cognitive impairment (MCI), 2) had chronic pain that the EMR or caregiver indicated was not well-controlled, 3) had a caregiver, according to informal assessment, who was both cognitively intact and agreeable to reserve the time to participate in the scheduled sessions (as assessed over the telephone by the recruiter), 4) lived with or had daily contact with the caregiver, and 5) had or could obtain the technology to participate (e.g., a smart phone, tablet, laptop, or desktop computer with audio and video capabilities; an email address for receiving video links; and dependable WiFi). If the dyad did not have an electronic device that would allow participation in video groups, they were loaned one by an established VA program.

The overarching goal of the project was to provide caregivers of older Veterans with chronic pain and dementia (or MCI) with a set of tools to help them: 1) recognize through behavioral observation when their loved one is experiencing pain-related suffering, as opposed to pain reporting; 2) effectively distract their loved one from focusing on pain; 3) develop their own coping strategies to help alleviate caregiver stress; and 4) communicate effectively with their loved one experiencing pain. At least one week prior to the first session, caregivers were mailed a packet that contained the participant workbook (a binder holding the Group TeleHealth Agreement, VA Veterans Health Administration Notice of Privacy Practices, and the main content and home practice assignments for each of the five sessions), the baseline questionnaires, and a stamped, addressed return envelope.

The intervention consisted of five 60–90-minute sessions over eight weeks using VA’s virtual platform, VA Video Connect (VVC). We selected a virtual delivery format to allow caregivers to participate from home, thus alleviating the stress of travel and having to arrange for care of the Veteran in their absence. Some dyads, but not all, had prior experience with VVC. The interventionists conducted test calls with participants and provided technical assistance before and during program implementation, as needed. The VA Office of Connected Care is available 24/7 and also assisted with test calls and technical assistance when needed. The first four sessions occurred weekly over four weeks. Session 5 was scheduled 3–4 weeks after Session 4, to allow time for practicing the skills learned and identify issues that occurred so they could be addressed at the final review session. Individual make-up sessions were scheduled if a caregiver and/or Veteran were unable to attend. Session-by-session content is summarized in Table 1. Each cohort consisted of two to five caregiver-Veteran dyads.

Sessions were conducted by two clinicians who had experience working with older adults and caregivers but not specifically in behavioral pain management. They were trained by a pain psychologist (LP) and a geropsychologist (KR) using a detailed treatment manual that had been developed for the original study (Porter et al. 2022). The manual was modified for the current project to accommodate the conduct of some intervention sessions using a caregiver group format. Sessions included training in four skills: pain severity measurement using the PAINAD scale (Warden et al. 2003) and/or pain thermometer (Herr 2011), brief relaxation, communication, and identifying, adapting, and scheduling pleasant activities using music as an example. Clinicians practiced sessions using roleplay prior to the conduct of the actual sessions with Veterans and caregivers.

As part of Session 3, the project lead (DKW) conducted a teaching session with caregivers about a rational approach to using pain medications that included: targeting pain-related suffering rather than pain reporting; the importance of treating pain comorbidities (e.g., depression, anxiety); treating pain pre-emptively in certain situations, e.g., prior to potentially painful activities; and a stepped care approach (e.g., starting with topical medications).

Training reinforcement of the treating clinicians was provided by the pain psychologist and geropsychologist who met with the treating clinicians periodically during the first two cohorts to answer questions related to group dynamics and to troubleshoot any other issues that arose.

### Data collected

The following measures were collected at baseline prior to the first intervention session. If participants did not complete the measures that were mailed to them (see above), one of the clinicians collected them by telephone.

1. Caregiver Measures
  - *Demographics*, including age, gender, ethnicity, race, education, and employment.
  - *Pain caregiving competence*, using four questions on a Likert scale developed by Pearlin and colleagues (Pearlin et al. 1990). For this project, staff asked caregivers to think specifically about how they feel when managing their loved one’s pain when answering the questions.
  - *Self-efficacy for managing pain*, using the 7-item pain management subscale of a standardized measure that assesses caregiver self-efficacy for helping the patient manage symptoms. Each item is a question that is rated on a 10–100 scale and then averaged (Lorig et al. 1989).
  - *Caregiver burden*, using the Zarit Burden Interview (ZBI) short form, a 12-item scale that asks questions, each rated on a 5-point Likert scale (Bédard et al. 2001).
  - *Depressive symptoms*, using the PHQ-9 (Kroenke et al. 2001).
  - *Anxiety symptoms*, using the GAD-7 (Spitzer et al. 2006).
  - *Engagement in self-care*, using the 9-item Caregiver Self-Care Practices Scale; each question is rated on a 0–4 Likert scale (Lee et al. 2019).
  - *Quality of life of the Veteran*, using the caregiver version of the 13-itemQuality of Life in Alzheimer’s Disease scale. Each question is rated on a 4-point Likert scale and results are expressed as a sum of the items (Logsdon et al. 2002).
2. Veteran Measures
  - *Demographics*, including age, gender, ethnicity, race, education, and employment.
  - *Quality of life*, using the 13-item Quality of Life in Alzheimer’s Disease scale (Logsdon et al. 2002). Caregivers were permitted to assist Veterans in completing this instrument.
  - *Dementia severity* was measured with the Functional Assessment Staging (FAST) scale by one of the geriatricians on the team (MR). Stage 1 on the FAST indicates normal cognitive function, stage 2 is MCI, and stages 3–4 is mild dementia. Stages 5–6 indicate moderate dementia, and stage 7 indicates severe dementia (Reisberg 1988).

Within 1.5–2 weeks of completing the fifth session of the program, all measures except for demographics and dementia severity were repeated. In addition, caregivers and Veterans were administered a Client Satisfaction Questionnaire (Nguyen et al. 1983). Caregivers also were asked to rank their preference (1 = most preferred choice to 4 = least preferred choice) for the program venue – in person, videoconference from local VA outpatient clinic, videoconference from home (used for the program described here), or telephone. We requested that post-tests be returned by mail in 8 to 14 days; we called those participants whose post-tests we did not receive by the stated date to remind them to complete their questionnaires and return them, or to complete them by phone if they preferred.

Semi-structured interviews were conducted by a qualitative methodologist (MH) after GEM clinical staff obtained verbal permission from caregivers to be contacted. Following permission for contact, the qualitative methodologist reached out by phone to caregivers that had completed the program to schedule a one-on-one telephonic interview. A semi-structured interview guide was developed with the input of the study team, which covered the following domains: general experiences with and feelings about caregiving, general experiences dealing with pain in the care recipient, how the care recipients’ Veteran identity affected caregiving, how rurality affected caregiving, thoughts and experiences on the intervention, and discussion of what aspects of the intervention were still in use and/or had been most meaningful for participants. Interviews typically lasted 30 minutes and were audio recorded.

### Data analysis

Data were analyzed using both quantitative and qualitative methodologies.

#### Quantitative analysis

We used means and standard deviations to summarize continuous variables and frequencies and percentages for categorical variables. Pre- to post-intervention change in continuous variables was assessed via descriptive statistics and paired samples *t*-tests. SAS^®^ version 9.4 (SAS Institute, Inc., Cary, North Carolina) was used for quantitative analyses.

#### Qualitative analysis

We took a Qualitative Description approach to this study to focus on participants’ experiences with the pain and coping skills program to understand participants’ thoughts and experiences without abstracting to the level of theory development, as is common in social sciences. Qualitative Description approaches are common in qualitative studies conducted in the health sciences (Sandelowski 2000, 2010; Kim et al. 2017). Qualitative interviewing and analysis were conducted by a single team member (MH) who was not affiliated with the design or conduct of the broader program in order to ensure that there was some separation between the study/implementation team and the qualitative evaluation. The qualitative expert (MH) reviewed the program materials and developed a semi-structured interview guide that asked about two main topics: (1) Caregiving experiences, including the experience of providing pain-related care in individuals with dementia, as well as how Veteran identity and urban/rural location affected caregiving; (2) Experience with the program, including which facets of the program were most liked and disliked, what they had learned that had proven relevant and useful, and any recommendations that they had for improvements. The guide was reviewed by the rest of the study team to ensure that all domains relevant to the study team were included. Interviews were conducted telephonically, with the interviewer calling out from Microsoft Teams.

Because this was a pilot study, we used common Rapid Qualitative Inquiry methods for data analysis (Beebe 2014) to ensure that feedback was both comprehensive and could be returned in a timely manner. Rapid approaches are commonly used in implementation science (Hamilton and Finley 2019). Following each interview, the interviewer summarized interview content. The interviewer (MH) checked the summaries against Microsoft Teams automatically-generated transcripts of the interviews for accuracy. She then reviewed and organized the summaries for the purposes of identifying commonalities and patterns in the interview data in a manner similar to the steps used in Conventional Content Analysis – e.g., review of data, organization of data, review of themes (Hsieh and Shannon 2005). To ensure that the single analyst had fully understood participant sentiments and analyzed them in a consistent way, resulting findings were discussed with the rest of the study team as a form of investigator triangulation. Questions asked by other study team members helped to refine and clarify qualitative findings. For example, the interviewer had initially collapsed findings related to two separate pain rating or interpretation scales, which other team members were able to differentiate using context clues.

## Results

### Quantitative results

Six cohorts comprising 20 dyads participated. Quantitative analysis was conducted for 17 that completed both pre- and post-tests. For 16 of the dyads, the Veteran and caregiver lived together and for one dyad they did not live together but there was daily in-person contact at the Veteran’s home multiple hours per day. Of the 20 initial dyads, 10 of the caregivers attended all sessions at the regularly scheduled time; 8 attended all sessions but some were makeup sessions; and 2 attended less than 5 sessions. Of the Veterans, who were expected to attend 2 dyad sessions, 12 attended both sessions at the regularly scheduled time; 4 attended both sessions but at least one of the sessions was a make-up session; 3 attended 1 session; and 1 did not attend either session. Caregivers missed regularly scheduled sessions because of difficulty with using audio or video technology, caregiver or Veteran illness, conflicting appointments, or forgetting the appointment time.

Baseline participant characteristics are shown in Table 2. Sixteen of 17 Veterans were male and all caregivers were female. All caregivers and 16 of 17 Veterans were white, non-Hispanic/non-Latino. One Veteran was American Indian or Alaska Native. All participants except one caregiver were at least high school graduates. Most participants were retired. On average, caregivers reported mild to moderate caregiver stress, mild depression, and mild anxiety. Veterans had, on average, mild dementia. Five participants had MCI.

Baseline to follow-up changes are shown in Table 3. Statistically significant improvement was observed for two measures, pain caregiving competence – specifically that they believe they have learned to deal with a very difficult situation; and self-efficacy for managing pain. None of the other measures demonstrated statistically significant improvement but descriptive statistics mostly trended in the anticipated direction except for quality of life. Client Satisfaction Questionnaire results are shown in Table 4.

### Qualitative results

Twenty-two caregivers were contacted, 20 of whom were part of the original cohort, and two of whom were participants in the clinical program that was continued after the completion of the current quality improvement project. Of 22 caregivers who were contacted, 10 ultimately completed interviews. (Seven did not respond to outreach, and five ultimately declined due to life circumstances.) Participants were asked about their experiences providing care for a Veteran with dementia, including how they dealt with pain in this context, as well as their experiences with the intervention. Findings related to these two domains are presented below. Participants were also asked about Veteran identity and the influence of rurality/urbanity on caregiving. Quotes related to each theme are presented in the Supplemental Table.

### Caregiving experiences

#### Finding 1: Pain can become more difficult to identify and treat as dementia progresses

Caregivers noted that pain, or how people respond to it, can intensify as dementia progresses, with Veterans no longer able to understand why they are in pain, and struggling to verbalize their feelings or follow pain relief instructions. Caregivers whose care recipients can verbalize their pain often reported an easier time providing care and relief, while those whose care recipients could not accurately describe what they were feeling described more difficulties recognizing and treating pain. Caregivers who could not relieve pain described feelings of frustration, helplessness, and compassion.

#### Finding 2: Caregivers’ lives are complex

Caregivers for older Veterans are generally older themselves and have exceedingly complex lives. Interviews for this study were conducted over a nearly 6-month period as caregivers often needed to reschedule interviews several times due to competing demands, including the care they provided and their own healthcare and social needs. Caregivers experienced health crises themselves, sometimes resulting in hospitalization, health crises involving other family members, and the deaths of family members, resulting in the need for emotional care but also complex processes like the settling of estates.

#### Finding 3: Caregiving in a familiar environment was regarded as essential and also contributed to rural participants experiencing their living environments positively

Participants were asked if they lived in rural areas, and to comment on how their living location affected their ability to provide care. While not all participants lived in rural areas (some were suburban), many caregivers noted that living in an environment that was familiar to the person with dementia was of vital importance, as it reduced confusion as dementia symptoms progressed. Participants also spoke positively of living on familiar streets, with known neighbors who looked out for their care recipients. Those in rural areas generally felt positively about their rural locations, both for their familiarity but also for the space and quiet that they provided, with some participants noting that rural areas with low traffic gave their care recipients more freedom to putter around on family land or in workshops in the backyard. Rural participants also felt well-served by the VA Healthcare System in their locations.

#### Finding 4: Veteran identity positively influenced both Veterans and caregivers

When asked about how Veteran identity affected the caregiving relationship, participants noted a positive relationship between Veteran identity and receiving care. This positive relationship had two facets. First, Veteran status provided access to healthcare and programming through the VA. VA healthcare was described as high-quality care, both for the Veteran, and for support systems provided to caregivers; it was also routinely described as highly accessible, even in rural areas. Second, participants described a “soldier on” or “suck it up” attitude in the Veterans for whom they provided care, which meant that they frequently did not wallow in or dwell on pain. Relatedly, some Veterans and their caregivers relied on the notion of “following orders,” either from physicians, or from the individual providing care, which ensured that Veterans followed instructions to the best of their ability.

### Intervention experiences

Overall, the intervention was very positively received, with participants reporting that they and/or the Veterans for whom they provide care looked forward to and benefited from the program. We identified 4 findings related to the intervention itself, described below.

#### Finding 1: Participants reported an increased understanding of dementia, resulting in increased patience while caregiving

The primary takeaway from the program was an increased understanding of dementia, including the experiences and thought processes of those who have dementia. Participants reported that behaviors that they used to view as frustrating or malicious on the part of their Veteran care recipients (such as not following instructions, asking repetitive questions, or difficulties remembering how to do things) are now reframed for them as symptoms of dementia. This deeper understanding included better ability to recognize non-verbal pain cues, for those whose care recipients could not verbalize their pain. Furthermore, deeper understanding resulted in increased patience with their care recipients, and with themselves. Breathing exercises that they learned were frequently described as helpful in allowing the caregiver to calm down and find a way to cope with the situation, rather than responding with anger. Participants also learned what they described as better ways to communicate given this new understanding of dementia, including the ability to communicate non-verbally (through touch, pictures, or shared activities), and to respond to questions from their care recipients with a simple yes or no answer, without launching into more complex explanations. Additionally, participants described benefiting from hearing about the experiences of other caregivers, as this reminded them that they were not alone, and gave them an idea of what changes might be coming for them in the future, in the event that another Veteran’s dementia was further progressed (or simply had different symptoms). This shift in understanding was very impactful for the participants.

#### Finding 2: Participants learned the importance of pleasant and self-care activities (for both the caregiver and care recipient)

In addition to learning more about dementia and resulting increased patience, participants frequently described learning the importance of pleasant activities (including self-care activities). These activities identified through the program (such as listening to music, dancing, light hobbies such as birdwatching, and breathing exercises) were regarded as soothing both for the Veterans and for the caregivers. They could also provide a sense of purpose and distraction. The program’s movement-based activities such as dancing were described by some participants as activities that they and their spouses had stopped doing at some point but enjoyed once reminded of them by the program.

#### Finding 3: The pain scales referenced in the program had mixed utility for caregivers

Participants had mixed experiences with the pain scales that were provided as part of the program. For participants who were able to trust that their care recipients’ ratings of pain were accurate and consistent relative to each other over time, the verbal pain rating scale could be useful in tracking pain, and/or in deciding between medication and behavioral pain management. However, others did not feel that the rating their care recipients gave was accurate because of inconsistencies in ratings, or a sense that the care recipient was simply pointing arbitrarily at a pain rating. Others found that their care recipient refused to use the scale. Participants also had access to a behavioral pain rating scale that was particularly useful for those whose care recipients could no longer verbally express pain. Those who did not find use for the behavioral pain scale felt that it was helpful to have on hand and anticipated it might be more useful in the future if their care recipient lost the ability to verbalize pain.

#### Finding 4: Participants identified several possible modifications to the program

Participants mentioned several possible modifications to the program. Primarily, these focused on building and maintaining a cohort of fellow caregivers, including the desire for larger groups of caregivers in the program so that they could hear a range of experiences (expressed by a participant who was in a smaller cohort), and the ability to maintain contact with fellow members of their cohort after the program ended. Additionally, the sole participant who was not the spouse of a Veteran receiving care requested modifications to consider other caregiving roles and relationships. Secondarily, several participants requested that the program provide information on where to go when considering placement of their care recipient in an assisted living facility, as well as information on how to make that decision, as several participants were struggling with such decisions at the time of the interview. Lastly, one participant requested the ability to have a recording of the breathing exercises.

## Discussion

A virtual clinical program designed to help caregivers of older Veterans with pain and dementia, cope with pain-related caregiving challenges, was well received. Preliminary evidence suggests that such a program could have significant impact on caregivers, particularly their self-efficacy for pain management. Almost all caregivers identified home videoconferencing as the preferred venue for participating, and they felt the duration of the program as a whole and the length of the sessions was appropriate. They most valued learning about dementia and having other participating caregivers to learn from. They also valued being reminded about the importance of scheduling pleasant activities and the breathing relaxation exercise.

While the number of caregiver-patient dyads who participated was modest, and the absence of a control group prevents drawing definitive conclusions, several findings may have encouraging implications for future clinical programs and related research. The program had a notable impact on caregiver self-efficacy, and others have highlighted the key role that self-efficacy plays in contributing to caregiver health and reducing caregiver burden/stress (Keefe et al. 2003; Phongtankuel et al. 2023). Future research on patients with pain and dementia (or MCI) should consider including potent interventions that target improving caregiver self-efficacy for managing pain.

It is noteworthy that even though the intervention focused on pain management, qualitative interviews indicated that participants most valued their learning about thoughts and behaviors in people living with dementia (Intervention Experiences, Finding 1), and found the teaching about pain measurement scales less valuable. Pain is a stressor and people who have chronic pain cope with it using cognitive skills (Turk 2004). Those with dementia have a decline in these skills, and their process of coping often is impaired. Such coping is applicable to stressors beyond physical pain. Thus, future programs may wish to consider more overt teaching about dementia and management of dementia-associated behaviors.

Having access to other caregivers who have similar experiences and challenges was also valued by participants. In fact, maintaining contact with other caregivers was suggested as a modification to future programs, not surprisingly given studies by others demonstrating the beneficial effects of caregiver support groups (Chien et al. 2011; Vandepitte et al. 2016; Oliver et al. 2017). Thus, including caregiver group sessions, as this program did, might be considered by professionals wishing to establish a program, along with facilitating contact among participants following program completion.

While group support was identified by participants as valuable, finding a mutually convenient time for offering group sessions can pose a challenge, given caregivers’ multiple responsibilities. Holding multiple sessions allows for concepts to be reinforced and strengthens the opportunity for participants to build relationships. However, despite almost unanimous agreement from participating caregivers that the length and number of sessions were “about right,” providers designing future programs might consider presenting fewer and/or shorter sessions in order to lower the barrier to entry. Future programs should aim to strike a balance between reduced time commitment and adequate exposure to learning and support. Fewer or shorter scheduled sessions could be supplemented by an online chatroom-style group to augment the support aspect of the program; these changes might allow participants somewhat greater flexibility in the times they participate and may improve ease of scheduling and enhance participation.

Program delivery was found to be feasible and well-received by caregivers. As noted, the program was delivered by healthcare professionals experienced in working with older adults, but without prior training in behavioral approaches to pain management. Clinicians required only brief training by a pain psychologist and a geropsychologist. Delivery using videoconferencing technology was the preferred venue by most participants. Videoconferencing also affords people with dementia the ability to receive the intervention in a familiar environment, an important feature highlighted by qualitative Finding 3 (Caregiving Experiences). These observations support the feasibility of disseminating such a program, that could be housed in many venues, depending on available resources.

While positively received overall, the program was not without its challenges, primarily scheduling and technological difficulties. The attendance data reflects the challenges of trying to schedule up to 5 participants simultaneously for 1½-hour sessions given their multiple competing responsibilities, such as being caregivers for multiple family members, having employment outside the home, or having multiple personal and/or family member medical appointments. These challenges were highlighted by the qualitative data (Caregiving Experiences, Finding 2). Additionally, lack of expertise with electronic devices (e.g., computers, tablets, cell phones) was a barrier for some, despite the tech support provided.

Researchers who wish to rigorously test the efficacy of programs such as ours should carefully consider some important questions. First, who are the targeted caregivers and how much do they already know about managing behavioral aspects of dementia? The perceived value and impact of a program that targets caregivers of older adults with pain and dementia could theoretically be influenced by caregivers’ expertise in dementia. Some dementia-associated behaviors such as perseveration and agitation are similar to pain behaviors, thus caregivers experienced in managing dementia-associated behaviors may have an advantage over those who are not. Veterans in our program were receiving care from VA geriatricians; thus their caregivers were somewhat experienced in dementia caregiving. Despite this, per the qualitative interviews, caregivers voiced appreciation of the knowledge gained about dementia (Intervention Experiences, Finding 1). Caregivers without such prior experience may have even more to gain. Future studies may wish to evaluate caregivers’ baseline knowledge and experience with managing dementia-associated behaviors, with an eye toward optimizing heterogeneity. Future studies should also consider including qualitative methods to understand the relationship between dementia stage and perceived value of teaching about managing dementia-associated behaviors versus evaluating and managing pain.

A related question that investigators should consider is, “What should be the case finding approach for potential study participants?” Dementia is underdiagnosed and under-documented in medical records (Amjad et al. 2018). Thus, recruiting participants by targeting those with an established diagnosis of dementia may restrict the potential participant pool. We recruited participants with an established diagnosis of MCI or dementia. In addition to recruiting participants from geriatrics clinics where dementia often is a focus of care, investigators may wish to identify potential participants by screening patients from primary care and pain clinics over a certain age, e.g. ≥ age 75, in whom the risk of dementia is significant (Caoa et al. 2020). While this approach is labor intensive, it could potentially optimize the likelihood of demonstrating the intervention’s efficacy.

Further, what should be the content of the intervention? Caregivers of older adults with pain and dementia often have complex needs that can impact their caregiving but that could be ameliorated, such as: practical aspects of caregiving, including but not limited to introduction of professional help into the home, or placement in an outside facility; the burden of caregiving itself; other mental health struggles (e.g., depression, anxiety); and physical illnesses. Veterans may have different responses to pain than non-Veterans (see Caregiving Experiences, Finding 4) and/or a greater prevalence of conditions such as post-traumatic stress disorder that impact their response to pain. Thus, future studies may wish to evaluate interventions that include not only teaching pain coping skills but also addressing individual caregiver needs, as well as individual patient needs, which may vary according to the stage of dementia.

Finally, investigators should consider outcomes that are relevant and likely to demonstrate change in response to the intervention. While self-efficacy may improve, literature published to date has included self-efficacy as a mediator/moderator variable rather than a primary outcome. Our clinical experience suggests that health care utilization may be an appropriate long-term outcome. That is, education about dementia in older patients with pain may ultimately prevent unnecessary procedures and medications that have potential toxicity (Panel 2023). The complex needs of these patients and their caregivers will become ever more pressing as our society continues to age and research to address these needs is urgent.

## Supplementary Material

Supplementary Material

**Supplementary material.** The supplementary material for this article can be found at https://doi.org/10.1017/S1478951526102144.

## Figures and Tables

**Table 1.** Intervention Content

Session	Content and Skills Taught	Attendees

1	Pain assessment	Caregiver group
	- PAINAD scale	
	- Pain thermometer	
	- Distinguishing pain reporting from suffering	
	Importance of Self-Care	

2	Pain coping skills training	Individual caregiver-Veteran dyad
	- Pleasant activity scheduling	
	- Relaxation exercise (breathing)	

3	Pain management strategies	Caregiver group
	- Relaxation review	
	- Supportive communication	
	- Coping with negative thoughts/calming self-statements	
	- Effective use of pharmacological strategies	

4	Skills practice	Individual caregiver-Veteran dyad
	- Relaxation and pleasant activities review	
	- Pleasant activities scheduling	
	Music for pain management	

5	Maintenance planning	Caregiver group
	- Review skills; Identify skills use obstacles and strategies to cope	
	Program feedback	

PAINAD: Pain Assessment in Advanced Dementia.

**Table 2.** Participant baseline characteristics [mean ± standard deviation or N (%)]

Characteristic	Caregiver (n = 17)	Veteran (n = 17)
Age (years)	73.7 ± 7.0	79.2 ± 6.1
Sex		
Female	17 (100.0)	1 (5.9)
Male	0 (0.0)	16 (94.1)
Race		
White	17 (100.0)	16 (94.1)
American Indian or Alaska Native	0 (0.0)	1 (5.9)
Ethnicity: Not Hispanic or Latino	17 (100.0)	17 (100.0)
Relationship of caregiver to Veteran		
Spouse	16 (94.1)	
Adult child	1 (5.9)	
Education		
No high school diploma	0 (0.0)	1 (5.9)
High school graduate or GED	10 (58.8)	8 (47.1)
Associate’s degree, academic program	1 (5.9)	2 (11.8)
Some college, no degree	3 (17.7)	2 (11.8)
Bachelor’s degree	1 (5.9)	3 (17.7)
Master’s degree	2 (11.8)	1 (5.9)
Employment		
Disabled due to pain	1 (5.9)	0 (0.0)
Retired	13 (76.5)	13 (76.5)
Employed as caregiver	1 (5.9)	0 (0.0)
Employed outside of home	1 (5.9)	0 (0.0)
Not reported	1 (5.9)	4 (23.5)
Pain caregiving competence (possible responses for each item: 1 to 4; higher is better)		–
How to deal with a very difficult situation	2.8 ± 0.8	
Feel that you’re a good caregiver	3.3 ± 0.6	
Competent	3.0 ± 0.7	
Self-confident	3.0 ± 0.7	
Pain self-efficacy (possible responses: average of seven items, each item 10 (Very Uncertain) to 100 (Certain) by 10s)	49.7 ± 17.3	–
Zarit Buren Interview short form (possible responses: 0 to 48 (0 to 4 per item on 12 items); higher indicates greater burden)	20 ± 9.3	–
PHQ – 9 (n = 14) (possible responses: 0 to 27 (0 to 3 per item on nine items); higher indicates greater severity of depressive symptoms)	9.7 ± 6.7	–
GAD – 7 (possible responses: 0 to 21 (0 to 3 per item on seven items); higher indicates greater severity of anxiety symptoms)	8.1 ± 6.7	–
Caregiver Self-Care Practices Scale (possible responses: 0 to 36 (0 to 4 per item on 9 items); higher indicates greater frequency of self-care activities)	19.1 ± 4.9	–
Dementia severity (FAST scale) (possible scores: 1 (normal adult) to 7 (severe dementia))	–	3.4 ± 1.2
Caregiver-assessed Veteran QOL (possible responses: 13 to 52 (1 to 4 per item on 13 items); higher is better)	28.6 ± 7.3	–
Veteran-assessed Veteran QOL (n = 12) (possible responses: 13 to 52 (1 to 4 per item on 13 items); higher is better)	–	29.7 ± 3.0

FAST: Functional Assessment Staging Tool; GAD-7: Generalized Anxiety Disorder 7-item scale; GED: General Educational Development; PHQ-9: Patient Health Questionnaire 9-item scale; QOL: Quality of Life.

**Table 3.** Changes from baseline

Variable	Baseline to Follow-Up Change Mean ± SD	95% Confidence Interval	*p*-Value

Pain caregiving competence			
- How to deal with a very difficult situation	0.6 ± 1.0	0.1, 1.2	0.0165
- Feel that all in all, you’re a good caregiver	0.1 ± 0.5	−0.1, 0.4	0.3322
- Competent	0.3 ± 0.8	−0.1, 0.7	0.1357
- Self-confident	0.1 ± 0.8	−0.3, 0.5	0.5434

Pain self-efficacy (n = 16)	18.8 ± 23.0	6.6,31.1	0.0051

Zarit Buren Interview short form (n = 14)*	−1.9 ± 5.8	−5.2,1.5	0.2535

PHQ – 9 (n = 14)*	−2.1 ± 5.2	−5.2,0.9	0.1504

GAD – 7*	−0.4 ± 5.4	−3.1, 2.4	0.7922

Caregiver Self-Care Practices Scale (n = 16)	0.5 ± 5.7	−2.5, 3.5	0.7307

Caregiver-assessed Veteran QOL (n = 15)	−1.1 ± 7.4	−5.2,3.0	0.5618

Veteran-assessed Veteran QOL (n = 6)	−6.2 ± 7.1	−13.6, 1.3	0.0870

GAD-7: Generalized Anxiety Disorder 7-item scale; PHQ-9: Patient Health Questionnaire 9-item scale; QOL: Quality of Life; SD = Standard deviation.

* Scale change in the negative direction indicates improvement.

**Table 4.** Caregiver Satisfaction (n = 20)

Question	Response Options	Caregiver Ratings (n)
How would you rate the quality of services you received through this program?	Excellent	12
	Good	4
	Fair	
	Poor	
Have the services you received helped you to deal more effectively with your family member’s pain?	Yes, they helped a great deal	9
	Yes, they helped somewhat	5
	No, they really didn’t help	1
	No, they seemed to make things worse	
Have the services you received helped you to deal more effectively with other caregiving responsibilities?	Yes, they helped a great deal	8
	Yes, they helped somewhat	7
	No, they really didn’t help	1
	No, they seemed to make things worse	
Would you recommend our program to a friend who is caring for someone with memory problems and pain?	Yes, definitely	11
	Yes, I think so	4
	No, I don’t think so	
	No, definitely not	1
How satisfied are you with the amount of help you have received?	Very satisfied	7
	Mostly satisfied	8
	Indifferent, or mildly dissatisfied	
	Quite dissatisfied	1
In an overall, general sense, how satisfied are you with the services you have received?	Very satisfied	7
	Mostly satisfied	6
	Indifferent, or mildly dissatisfied	
	Quite dissatisfied	4
Did you think five sessions was:	Too few	2
	About right	15
	Too many	
Did you think the length of the sessions was:	Too short	
	About right	17
	Too long	
Given a choice on how to participate in this type of program, how would you **rank** the options? (“most preferred”)	In person	1
	Videoconference from local VA outpatient clinic	1
	Videoconference from home	15
	Telephone	
How convenient was it for you to participate in this program over videoconference?	Very convenient	14
	Mostly convenient	2
	A little inconvenient	
	Very inconvenient	1
