# Supplementary material for: A virtual program to teach pain coping skills to dyads of caregivers and Veterans with dementia or mild cognitive impairment: Preliminary quantitative and qualitative findings
Source: Palliat Support Care. Author manuscript; Available in PMC 2026 May 11. (PMC13159481; doi:10.1017/S1478951526102144)
Supplement: Supplementary file 1 [file NIHMS2164654-supplement-Supplementary_Material.docx]

| **Caregiving Experience Themes** |
| --- |
| *Finding 1: Pain can become more difficult to identify and treat as dementia progresses.* |
| Most of the time I have to recognize it [that he’s in pain].  Once in a while, he does say something. He's up and down with his dementia. Some days he doesn't know what's going on. I mean, it's like, Oh my gosh. And then some days he's really clear. (Participant 28) |
| I can tell by the expressions on his face, if he is in pain, or if he's just not paying attention to something, or… I think mainly it's by the expression on his face. You know, I'll have to ask him, what's wrong? Do you need some Tylenol or what's going on? You know, I'll try to get it out of him. What's going on? But sometimes he just, with the dementia getting a little worse, [it’s] harder, because he just says I don't know. So I don't know. (Participant 7) |
| I knew he was in pain [before the dementia] when that he had back problems and I think problems with his knees. I knew that it was pain [that made him act the way he did] and sometimes I would have to still have to, you know, leave the room for a little bit [if he was shouting or swearing because of pain]. I just leave the room or find something to do and that really bothers him because he wants me there with him a lot. […] He's always been that way, but [since the dementia] it has really, really increased. (Participant 4) |
| *Finding 2: Caregivers’ lives are complex, highlighting both the need for support and the demands of providing care.* |
| Well, it's [the program] given me a support network, if I need it, I mean there's numbers [to call]. I'm getting messages, I get newsletters. If our personal lives would settle down. We’ve not had we've not had the best year and so I haven't been able to access any of those but in the future I may. But right now [due to my mother’s death and unexpected house maintenance] I'm we're messed up in a big way. […]I'm telling you, we have had a horrible year. Horrible. (Participant 29) |
| We've had enormous health problems and so we've been dealing with all of that at the same time, he's at the point now where we cannot leave him alone, period. If I have to go somewhere, I have to import one of our family members or whatever to allow me to go. I take him everywhere with me usually. But there are times where I can't take him, […] so when those occasions arise, I get [one of my family members to] stay with him. Or I tried to make the appointments when the caretaker's here so that I can go and get back. (Participant 27) |
| *Finding 3: Caregiving in a familiar environment was regarded as essential and also contributed to rural participants’ experiencing their living environments positively.* |
| If I if I lived in the city with him, it would not work. He is very he's very busy. We have almost 13 acres and he has a shed and he can go to and from his shed now and he can dig around, which that was the whole thing. (Participant 28) |
| We have really good neighbors that if I needed anything, they'll help me and I guess you know… But we had a deck put on the house for him so that we could at least sit out back and and be comfortable. And you know, just try to do everything to make him comfortable, which inadvertently helps me too. (Participant 24) |
| *Finding 4: Veteran identity positively influenced both Veterans and caregivers.* |
| He's definitely a soldier on kind of guy. Like you just put some dirt on and go. To that's him. He's not gonna sit there and whine about it. He's he'll just soldier on and he and. And that is a term that he uses. […] I'm a soldier on kind of person too, mostly. The more we dwell on it, the worse it gets. (Participant 28) |
| Well, basically, we just suck it up and get the job done. What if whatever he has to have done, make sure it gets done. I do his medicine. appointments. I go in with him. Yeah, he he participates as much as he can. And some days, that's good. Other days he he doesn't even know why he's there. (Participant 29) |
| **Intervention Experience Themes** |
| *Finding 1: Participants report an increased understanding of dementia, resulting in increased patience while caregiving.* |
| I'm a patient person, but sometimes when you're dealing with a person with dementia, it takes a lot more patience. And [the program] helped me know [that] I wasn't perfect in my thinking of how things should be done as to how they can be done, that is. Basically, one thing I came away with was that. [Now] I don't push for it to be done exactly when I think it should be done, that maybe it can be done at another time, unless you know it would be an emergency type situation, but it can wait if it can't be done right now. (Participant 15) |
| I think it [the program] makes you look at the other perspective. That's what I really think it does. It makes you understanding more to look at somebody and see what they're going through because you never know what's on someone else's mind or what they're going through. And just to be able to look at his face and know he's in pain or know what he needs. Is it's very helpful to me and then to help him, you know, just like showing him pictures or dancing with them or anything like that. […] He has a hard time talking, and so if I can show him another way, I think that's really beneficial. (Participant 24) |
| I'm trying to think of something [I learned from the program] that's happened lately. I’m still trying to do this: just answer yes or no. If I get into details on why I did this or why I did that or whatever he doesn't want to hear it. He just, he said, just answer yes or no, and I'm still working on that. And because that really agitates him. […] That was something that really did help because I was trying to explain things to him, like I did my children. […] Well, he doesn't wanna hear that. He just says, “no or yes, you don't have to get in and tell me anything else.” (Participant 4) |
| I use [the breathing] all the time actually. Well, when I'm about to flip my lid because I just can't take the stress anymore, I have to walk away and breathe. And believe it or not, I mean it just calms you down so quick. And it's like, OK. Now I can go back in here and see what he's gonna do next to really raise my blood pressure. (Participant 27) |
| Hearing that the other ladies were going through something similar was nice to know. Hey, you're not alone. That, you know, other people are going through this so. Wish I wasn't, but it was nice to know that. There was other people going through it and trying, you know, to get help. Like I'm not a help me type person but this program did show me that you know it was OK to accept some help and to listen to it. (Participant 15) |
| *Finding 2: Participants learned the importance of pleasant and self-care activities (for both the caregiver and care recipient).* |
| The breathing exercises I like 'cause. I've done that. But he he doesn't get it. I don't know. He just he just doesn't get that. But he likes music. I mean, he'll watch something on TV, like the voice or American Idol or anything with songs and music. He does like music and that will distract him. (Participant 7) |
| Well, I love to do yard work in the summertime. I'm always out and I'd say, well, you know, why don't you sit out here on the porch and you can, you know, advise me a little bit. […] And he'd get a kick out of me because I like to feed the birds and talk to the birds. So once in a while he would get up in the morning and start filling the bird feeders and the water dishes. [It helps him] feel like [he has] a purpose. I think it's the best way I could describe it, is that you got to make sure that they don't feel useless. (Participant 15) |
| I like the idea of how to measure the pain and what to do because then, instead of me jumping in to hurry up and giving a pill, you know, a couple Tylenol or something, [instead] I could just talk to him and maybe turn the stimulator on for him to help him. Or just, listen to some music to take his mind off of it. Something like that, which was good. I actually got him to listen to [some classical music] he actually said then, Come on, let's get up and dance. He's not a dancer, but we got up and just rocked back and forth, and […] he even enjoyed it and it helped him, I think, to forget about the pain a little too, just to get his mind on to something else. (Participant 24) |
| *Finding 3: The pain scales referenced in the program had mixed utility for caregivers.* |
| I mean, it's hard to tell on the pain scale. [Veteran name] always says he's an 8. I said he can't be an 8. You won't be able to fall asleep so easily if you are an 8. I don't think he understands the pain management like the scale. […] To me when I'm in pain. I mean, I've had hip replacements and a knee replacement. I can't sleep, but he manages to, so I don't know. (Participant 7) |
| And I did use like the pain scale. I tried to at least cause back. Then again, he was so confused in that. You know what I mean? Where like you know, I needed that. But again, now he can just tell me what bothers him and stuff? (Participant 9) |
| We tried to use a couple of those things. He's not very cooperative, but you know, we just did the best we could with it. “What's your pain on this scale” from whatever. And so he would say, “OK. It's right here.” OK. So then an hour later, after you give him some Tylenol and you say, well, “how is your pain now? Is it better or is it? You know, how is it?” “Oh, it's a lot better.” “OK, on this scale. Where would you put it?” And he'll do a higher pain number. So I don't know. (Participant 27) |
| So the pain scale I do when he comes out for breakfast, you know, I make sure he gets his breakfast and his meds and everything. And that's usually when I do my assessment. Also at night, he's not a big pill taker, so I have to try to figure out at night, you know, if he's having pain, ‘cause then I got to give him the Tylenol, which he usually doesn't want to take, because he doesn't like to take medication or vitamins or pills or anything. (Participant 28) |
| *Finding 4: Participants identified several possible modifications to the program* |
| That's really an awkward thing [massage, dance, or physical contact with her step-father, the Veteran with whom she completed the program]. […] Yeah, we don't have a lot of physical interaction. Yeah. (Participant 28) |
| I think it should cover like when it comes to a time where you can't take care of somebody. What do you do? You know, where do you turn to? What are all the possibilities that you can like, [are] there community nursing homes that would take care of, which I didn't know that I found out when I talked to somebody, but I think they should talk about that more. (Participant 7) |
| I'd like for it [the group interaction]  to continue. [Either] on the computer or maybe just meet up somewhere we don't have to give our names or anything, but just to meet up somewhere. And see how they're going. Some of the women, their husbands were further along than mine and that that was a good insight on what I may have to look forward to. And so that that was a big help, but. And I liked sharing things.  (Participant 4) |

Selected quotes are presented here by theme (although readers may observe some overlap in content, in which a quote presented as exemplary of one theme also contains information related to another theme). We have endeavored to quote from as many participants as possible, noting that there is natural variation in the level of detail provided by different participants that makes some more “quotable” than others, in that their quotes are more informative, particularly when standing alone outside of the context of an entire transcript. Quotes have been minorly edited for readability, with repeated words removed. Any redactions beyond the removal of repeated words are marked with […]
